# Consistency and flexibility of character in free-ranging male African elephants across time, age, and social contexts

**DOI:** 10.1371/journal.pone.0311780

**Published:** 2024-12-04

**Authors:** Caitlin E. O’Connell-Rodwell, Jodie L. Berezin, Colleen Kinzley, Patrick T. Freeman, Monica N. Sandri, Dustin Kieschnick, Timothy C. Rodwell, Mariana Abarca, Virginia Hayssen

**Affiliations:** 1 Center for Conservation Biology, Stanford University, Stanford, CA, United States of America; 2 Harvard University Center for the Environment, Cambridge, MA, United States of America; 3 Utopia Scientific, San Diego, CA, United States of America; 4 Department of Biological Sciences, Clark Science Center, Smith College, Northampton, MA, United States of America; 5 Conservation Society of California, Oakland Zoo, Oakland, CA, United States of America; 6 Conservation Science Partners, Truckee, CA, United States of America; 7 Geography Graduate Group, University of California Davis, Davis, CA, United States of America; 8 Weill Institute for Neurosciences, University of California at San Francisco, San Francisco, CA, United States of America; 9 School of Medicine, University of California San Diego, La Jolla, CA, United States of America; University of Mississippi School of Pharmacy, UNITED STATES OF AMERICA

## Abstract

Post-dispersal male African elephants (*Loxodonta africana*) live within complex social networks. To quantify the consistency of male elephant character (or personality) within these networks, we employed behavioral repeatability analysis tools across social and environmental contexts. We collected behavioral data from thirty-four individually-identified male elephants at the same waterhole over five field seasons (2007–2011) in Etosha National Park, Namibia. Using repeatability models to assess ten behavioral categories, we found five behaviors (affiliation, aggression, dominance, self-directed anxious, and self-directed comfort) were consistent at the individual level. Some of these behaviors were also repeatable, depending on social context. In particular, the presence of younger males and a keystone male (i.e., the most dominant and socially-integrated individual during our study period) had the biggest impact on adult male behaviors. Surprisingly, the presence of elephants in musth had little impact. Finally, we found that younger individuals were more alike in their overall character profiles than older males, further supporting the hypothesis that male elephants develop unique, yet socially-flexible character types as they age. These results demonstrate that male elephants possess distinct character traits that are also behaviorally adaptable, depending on the social context. Overall, our research further highlights the complexity of male elephant individuality and social dynamics that might be leveraged to improve in-situ and ex-situ management and conservation decisions for the species.

## Introduction

Individual social animals within a population often express certain behaviors—or sets of correlated behaviors—differentially, and may do so consistently or flexibly across time, space, and environmental gradients [1–4]. More recently, the field of Conservation Behavior has emerged in an effort to understand which behaviors have important implications for the conservation of vulnerable species, as well as the mechanisms that enable some animals to succeed or fail to adapt in a rapidly changing world [5]. However, given the ubiquity and diversity of behaviors present across taxa, scientists have the challenging task of determining which species, demographic groups (e.g., juveniles or adults), behavioral traits (e.g., aggression or affiliation), or study contexts should be prioritized [5]. Likewise, researchers must also decide how behaviors should be measured in order to best inform wildlife management and conservation policies.

The African savannah elephant (*Loxodonta africana*) is a highly intelligent, socially complex, and long-lived megafaunal species that has been a conservation priority over the last several decades. Like other large mammalian herbivores, elephants provide key ecosystem services [6] and are both culturally and economically significant, yet are severely impacted by anthropogenic disturbance and climate change [7]. One way to improve conservation efforts is through behavioral repeatability research, repeatability of behavior being a proxy for animal character (often referred to as personality, temperament, or consistent individual differences) at the population level [1, 2]. For example, a behavior like boldness, a measure of an individual’s reaction to a new or risky situation [8], might inform conservation managers of which elephants are likely to or are engaging in human-elephant conflict, and a behavior such as exploration, an individual’s reaction to a novel situation or object [8], can aid in the creation of mitigation measures that are specific to individuals who repeatedly engage in human-elephant conflict.

To date, all three species of elephants are known to display behavioral consistency. However, the current body of research on elephant character is based largely on captive [9–16] and semi-captive elephants [17–20], with male data being grouped with female data, and only two studies on wild elephants to date (*L*. *cyclotis—*[21]; *L*. *africana*—[22]). Amongst studies, the most common character traits reported were ‘sociability’ and ‘aggressiveness’. Additionally, captive and semi-captive elephants were defined more by traits related to nervousness and interactions with caregivers, such as attentiveness and cooperation, while wild female elephants were defined more by leadership or confidence. While these previous studies provide the foundation for elephant character research, their results might not be easily generalizable to free-ranging systems, given the inconsistencies between the captive, semi-captive, and free-ranging environmental contexts.

In addition, research on elephant character and behavioral consistency have primarily focused on females. Unlike female elephants who spend their entire lives in family groups, males navigate complex all-male societies and spend more time alone than their female counterparts due to sex-based differences in reproductive strategies [23]. Male elephant society is dynamic, consisting of dominance hierarchies [24], complex social networks and associations [24–28] based on long-term relationships [27], kinship [25], age structure [26, 29, 30], and reproductive status [31, 32]. These differences in life history likely impact the expression of consistent behaviors. The lack of research on male elephants is likely due to the limited sample sizes in captivity, as well as the challenge of collecting long-term, individual-based behavioral observations for a highly mobile species in the wild.

The goal of this study was to determine whether free-ranging male elephants of post-dispersal age differentially express behaviors consistently and/or flexibly as a function of time, age class, and social context. Using a long-term behavioral dataset collected from individually-identified male elephants in Etosha National Park, Namibia, we first quantified the repeatability (or consistency) of ten behavioral categories in order to establish the most stable character traits in our study system. We expected at least some of these behaviors would be repeatable based on their significance in previous elephant character studies, such as affiliation and aggression, and some to be more flexible to cope with a dynamic environment. Our second aim was to determine which social contexts (i.e., presence of younger males, males in musth, or a keystone individual) explain the variation observed in character traits. Based on the social dynamics of male elephants and long-term observations at the waterhole, we hypothesized that these social contexts would illicit behavioral flexibility in varying degrees for each behavior, dependent on both the behavior and the context (e.g., aggression being heightened when musth bulls are present). Finally, our third aim was to test for age-based similarities and differences in male elephant character profiles to determine possible developmental patterns and effects of group cohesion on elephant behavior. We predicted there would be similarities in character within age classes due to similar social and environmental needs.

## Methods

### Study site

As part of a long-term elephant monitoring project that started in 1992, behavioral observations were collected on male elephants at the Mushara waterhole (hereafter Mushara) in the northeastern corner of Etosha National Park (ENP), Namibia from 2005–2011. We chose a 5-year subset of this data to analyze, collected from 2007–2011, during 6-week field seasons from mid-June to the end of July for each consecutive year. This subset of data contained the most consistent information on the selected study subjects relevant to this research question. ENP is a fenced park that encompasses 22,970 km^2^ [33] and supports an elephant population of approximately 2,400 individuals [34]. The Mushara waterhole is fed by a permanent, artisanal spring and is the only source of drinking water within a 10 km radius [35]. The waterhole is situated within a 0.22 km^2^ clearing [36]. Behavioral observations were collected from an 8-meter-tall research tower situated about 80 meters north of the waterhole with a 360-degree view of the clearing. Elephants enter the clearing from eight well-traveled paths from the brush and usually walk directly to the water trough or pan (see [36, 37]). Water flows from the source of the spring into a trough, the head of which has the freshest water and is the preferred location to drink.

All data collected was purely observational. This study was conducted according to the guidelines of the Declaration of Helsinki and approved by the Namibian Ministry of Environment and Tourism. Field permit numbers for study years are available upon reasonable request.

### Elephant identification and age classification

Many elephants that visit Mushara have been individually identified and tracked across years using morphological traits such as ear-tear patterns, tail-hair configuration, tusk size and shape, and body size [24, 38]. We assigned male elephants a relative age class based on shoulder height, hind foot length, and facial characteristics [39]. Age classes include: one-quarter (1Q), 10–14 years old; two-quarter (2Q), 15–24 years old; three-quarter (3Q), 25–34 years old; full, 35–49 years old; and elder, 50 years and older.

We included a total of 34, independent, non-musth males as the focal subjects in this study, while the presence of musth males was considered a social context (see the “Social context descriptions” section below for more details). All focal subjects were independent adults and not observed as part of natal herds. We chose individuals who were observed at least three times across two years (mean occurrence per individual = 15.4 ± 10.0, range = 4, 40), with a mean of 21 individuals observed per year and a range of 14 to 28 individuals (S1 Table). Five males changed age classes during their observation years [39]. Since age classes for males span approximately 10 years, we did not anticipate abrupt shifts in the expression of behaviors observed in these five individuals. As such, we categorized these individuals as the age class they remained within for most of the study (e.g., 4 out of 5 years). For the 34 males, four were classified as 1Q, six were 2Q, nine were 3Q, twelve were full, and three were elders.

### Behavioral observations

Elephant behavioral observations were recorded from approximately 11:00 am to 5:00 pm when elephants are visible and easily distinguishable. We collected behavioral data as “events”, where an event began when one or more elephants entered the clearing and ended when all elephants left the clearing. We terminated an event at 15 minutes if an elephant was alone at the waterhole. Since the focus was on male elephants, we removed events with female elephants. In cases where females arrived during an event, we kept the part of the event before the females arrived. We collected behavioral observations using all-occurrence sampling [40] for all elephants present at the waterhole using a customized datalogger programmed with Noldus Observer software (Noldus Information Technology Inc., Virginia, USA).

We recorded sixty-six distinct behaviors (Table 1; S2 Table). To include the full suite of unique behaviors that male elephants display and to avoid removing low-occurrence behaviors, we pooled behaviors with a similar context (or expressions of similar behavioral patterns) into ten categories [24, 41]. The fine-scale behaviors in each category and category definitions are listed in Table 1, while definitions for the fine-scale behaviors are provided in S2 Table. Moving forward, these behavioral categories will be referred to simply as “behaviors.” The final ten behaviors include affiliation, aggression, displacement, escalated aggression, play, retreat, self-directed anxious, self-directed comfort, social contentment, and vigilance.

**Table 1:** Condensed ethogram describing the ten behavioral categories and list of sixty-six discrete behaviors that make up the behavioral categories.

Behavior (category)	n	Behavior category definition	Fine-scale behaviors
Affiliation	2,461	Positive interaction between individuals; aids in relationship building and maintenance.	Backs into; Body to body; Ear on face; Ear on rear; Follows; Foot to body; Head to body; Head to head; Inspecting; Mount; Pre-mount; Pushes; Rubs; Tail to body; Trunk to body; Trunk to head; Trunk to mouth; Trunk to temporal; Trunk to tusk; Trunk wrap
Aggression	2,038	Negative interaction to intimidate or threaten other elephants; no physical contact made between elephants.	Aggressive ear flap; Ear fold; Ears held out; Foot toss; Hard ear flap; Head held up; Head shake; Open mouth threat; Tail slap; Trunk drag; Trunk fist; Trunk throw
Dominance	872	Displacement of another individual; used to calculate the dominance hierarchy.	Displacement
Escalated aggression	69	Negative interaction to threaten or attack other elephants; often no physical contact.	Charge; Chase; Combat; Head down; Head down; Head thrust; Lunge; Pursue; Stand off
Play	493	Social play where individuals engage in exaggerated or loose movements and friendly sparring that avoids injury.	Gentle sparring; trunk swing
Retreat	206	Avoidance of aggression from another individual.	Back up; Retreat
Self-directed anxious	1,882	Nervous and reactive behaviors directed towards oneself; often to attend to the actions of other elephants or after receiving aggression from another.	Foot rubs; Tail lift; Touches own tusk; Trunk own mouth; Trunk own temporal; Trunk suck; Trunk twist
Self-directed comfort	498	Comfort or relaxed behaviors directed towards oneself.	Cross leg; Rest trunk; tusk hang
Social contentment	1,992	Group activity; relaxed behaviors observed during times of stillness at the waterhole.	Ear flap; Tail swing
Vigilance	4,616	Environmental assessment, often towards a conspecific to attend to the actions of other elephants; vigilance behaviors directed toward human activity were removed.	Freeze; Freeze trunk ground; Look; Orient; Over shoulder; Smell

Behaviors and categories are adapted and modified from [24, 41]. For behavior categories, n represents the total occurrence of discrete behaviors within that category, pooled across individuals, events, and years.

### Identifying the keystone individual

We wanted to examine the degree to which the presence of key social actors within the population may affect behavioral repeatability. For this social context, we identified a keystone individual based on two facets of social importance: 1) social network centrality and 2) dominance rank. Prior to network and dominance analyses, we filtered the dataset to (1) remove all individuals without a positive identification (i.e., unknown individuals), (2) remove all individuals recorded as being in musth during the observation year, and (3) remove all positively-identified individuals that were recorded at fewer than three independent observation sessions (events) during the season.

1. *Social network centrality*: For each year, we constructed association networks based on co-presence at the waterhole during observation sessions. For the purposes of our analyses, we applied the Gambit-of-the-Group approach [42], assuming that all individuals recorded during the same observation session were associating with each other, even if they arrived and/or departed at different times.
  For each year, we built weighted matrices of dyad-level association indices based on the Simple Ratio Index of association (SRI; [43, 44]) with larger association indices indicating individuals were more closely associated, and from there, calculated individual eigenvector centrality scores. This network metric is frequently used to quantify an individual’s influence on the broader network by assessing both its direct connections and the connections of its neighbors. An animal is considered more central if connected to other individuals that are also well-connected in the social network [45]. This metric helps identify key individuals that may play influential roles in information flow or social dynamics within the animal group. Networks and association indices were calculated using the *asnipe* package [46] and eigenvector centrality was calculated using the *igraph* package [47].
2. *Social dominance hierarchy*: In this study system, the displacement of an individual—defined as an instance where one elephant forces another to change his position and move away, possibly so that the initiator can take the position [24]—is an obvious non-combat behavior used to express dominance. We used dyad-level displacement contests to construct an ordinal dominance hierarchy per year and identify the most dominant individual in the population.
  We calculated ordinal dominance hierarchies using the normalized David’s Score (DS). DS estimates an individual’s dominance by considering the proportion of an individual’s dominance interactions that result in wins or losses across all the dyads with whom he interacts while considering the total number of dominance interactions observed. The highest values are assigned to individuals that most consistently win their contests [48–50]. Raw DS are converted to normalized David’s scores, such that in a population of N individuals, scores vary between 0 and N-1, with the highest value identifying the most dominant individual in the defined population (see [49] for derivation). Displacement contest matrices were constructed using the *Perc* package [51] and normalized DS were calculated using the *EloRating* package [52].

### Social context descriptions

We assessed behavioral flexibility across different social contexts at the waterhole. The waterhole acts as both an important resource and a place where a large suite of social interactions among elephants can be easily observed. We chose three social contexts that occurred frequently at Mushara waterhole and that we hypothesized, based on long-term observations, appear to influence male behavior: the presence of a musth male, the presence of a keystone male, or the presence of young males.

Since overlap in the three social contexts occurred frequently, we defined a total of eight social contexts in the study. First, we used presence-absence coding to categorize each event into the three social contexts; the presence of musth males, the keystone male, and young males (defined as individuals in the 1Q and 2Q age classes). Then, to account for events with an overlap of social contexts (e.g., musth and young males present), we included an additional four contexts. The eighth and final category was events with only adult, non-musth males (age classes 3Q, full, and elder). This category was considered the baseline social context and labeled as “adults only.” Due to the low occurrence of events where both a musth male and the keystone male were present (n = 1), as well as the musth male, keystone male, and young males were present (n = 2), we removed these events from subsequent analyses, leaving six total social contexts (Table 2).

**Table 2:** Social context categories, descriptions, and the total interval occurrences.

Social context category	Description	Total occurrences
Musth	Musth male(s) present with other males in the older age classes. Young males and the keystone are not present.	21
Keystone	The keystone male is present with other males in the older age classes. Musth and young males are not present.	13
Young	Young males present with or without older males. The keystone male and musth males are not present.	55
Musth and young	Musth and young males present with or without older males. The keystone male is not present.	14
Keystone and young	Keystone male and young males are present, with or without older males. Musth males are not present.	26
Adults only	Only older males (3Q+) are present. Musth, keystone, and young males are not present.	68

Young males are those in the 1Q and 2Q age classes and older males (“adults”) are those in the 3Q, full, and elder age classes. The social contexts of musth male and keystone male (n = 1), and musth male, keystone male, and young males (n = 2) are not displayed here due to low sample sizes.

### Intervals for changing social contexts

The social context often changed during long events. To account for this, we assigned an interval each time the social context changed within an event. As an example, some adults and young males were present at the start of the event and the interval was labeled as “1.” Then, the keystone male arrived, so the first interval of the event ended, and the second interval began. We removed intervals with solitary elephants, family groups, or only unknown males. Across the five years, this left a total of 148 events (mean per year ± SD: 29.6 ± 10.41) that contained 200 intervals (40.0 ± 13.96) (S1 Table). The mean time per event was 70.14 ± 47.10 minutes (range = 6.95, 249.39) and the average time per interval was 49.61 ± 33.76 minutes (range = 5.19, 178.72).

### Statistical analysis

All statistical analyses were conducted using R (version 4.3.1) [53]. Significance was evaluated at α = 0.05 for all models.

#### Repeatability analysis

We used repeatability models to assess the consistency of elephant behaviors. Repeatability (*R*) is an intra-class correlation measure that is used in animal personality research to quantify stable individual differences in behavior at the population level [54]. Repeatability models use the mixed-effects model framework, where the random effect estimates variance of repeated behavior measures of individuals. *R* is calculated as the group-level variance over the sum of the group-level and residual variances. In other words, where low intra-individual variation and high inter-individual variation creates a high repeatability, or *R* value [2].

We calculated behavior rates per year per elephant as the frequency of each behavior within an event over the total time the individual was present in that event. We then transformed the rates using the natural log due to significant right skew for each of the ten behaviors. We removed individuals that had less than two rates for a given behavior.

We fit a total of 10 models using the ‘rpt’ function in the *rptR* package [54]. We fit the models with the ln-transformed behavior rate as the response variable, with crossed, random effects of year, event, and individual ID. Year and event were included as random effects to account for sampling structure. Additionally, we calculated the repeatabilities for year and event to evaluate the impact that time (year) and social context (event) has on behavior rates, where significant repeatabilities indicate that behavior rates were stable within events/years and differ amongst events/years. We specified each model with a Gaussian distribution and 1000 bootstraps for the calculation of confidence intervals and *p*-values. We inspected all model residuals to confirm assumptions of homoscedasticity and normality of residuals were met.

The rptR package calculates confidence intervals using parametric bootstrapping and *p*-values using likelihood ratio testing, leading to conflicting results such as significant *p*-values and confidence intervals containing zero [55, 56]. In addition, repeatability values reported in nonhuman animals are typically low [2]. As such, we determined whether behaviors were repeatable using a combination of the *R* value, confidence intervals, and *p-*values [56]. We considered behaviors repeatable if the *R* value was greater than 0.1, did not contain 0 in the confidence interval, and had a significant *p*-value.

#### Behavioral flexibility among social contexts

We used linear mixed-effects models to assess the impact of social context on behaviors that had significant event repeatability. For this analysis, we focused only on the rates observed for adults (age classes 3Q, full, and elder) and removed the rates for young males (age classes 1Q and 2Q) and the keystone male. This method allowed for a direct comparison of how adults change their behavior rates in social contexts and removed the impact that the keystone male or young males have on behavior rates. For this analysis, we re-calculated behavior rates at the interval level (see above, “Intervals for changing social contexts”), rather than the event level (as was done for the repeatability analysis) to measure the impact that social context had on adult male elephant behavioral consistency.

We fit a model for each of the behaviors with a high event repeatability, with behavior rate as the response variable and social context as the fixed effect. Nested random-effects of year, event, and interval were included, as well as a partially-crossed random effect of individual ID to account for repeated measures of individuals. We transformed behavior rates using the natural log to meet normality assumptions. Models were fit using the ‘lmer’ function in the *lmerTest* R package [57]. This package builds on the lme4 package by including denominator degrees of freedom and *p*-values estimated by Satterthwaite’s method. Estimated marginal means were calculated for each model using the ‘ggpredict’ function in the *ggeffects* R package [58]. Model assumptions of linearity, homoscedasticity, and normality were assessed using functions from the R package *DHARMa* [59]; assumptions were met for all models.

#### Age related differences in character profiles

To assess the similarities of the character profiles among individuals, we used a non-metric multidimensional scaling (NMDS) clustering analysis. We used NMDS clustering since the behavior rate data was not suitable for traditional, personality data-reduction techniques (e.g., factor or principal components analysis) due to the correlation matrix failing to meet the Kaiser-Meyer-Olkin (KMO) and Bartlett’s test of sphericity criterion [60]. NMDS clustering is typically used for assessing similarities in species compositions among communities, where the number of individuals of each species is recorded per site [61]. An important assumption is that sampling effort is uniform across sites, so the abundances of species between sites are directly comparable. Due to differences in occurrence (total time each individual was observed) among individuals, count data were not appropriate, and proportions were required. For each individual, we calculated the proportion they displayed each repeatable-by-individual behavior, which we’ve termed an individual’s character profile.

Since proportions are compositional data, characterized as being bound by a lower and upper limit and existing in ‘simplex’ space, the data need to be transformed to be brought into ‘real’ space using the Aitchison’s distance [62]. Aitchison’s distance is calculated by transforming compositional data using the centered log-ratio (clr) and then calculating the Euclidean distance [63, 64]. A modified version of the clr transformation was used, called the robust clr [65] to account for individuals who do not display one of the behaviors (n = 7). The robust clr allows for “true zeros” to remain in the dataset without affecting downstream analyses or removing the individuals who did not display a behavior [65]. We considered the lack of these behaviors as a true zero, since the elephants did not display these behaviors during the observation time. As such, we performed data transformations on all nonzero values. We then calculated the Euclidean distance matrix with the robust clr transformed data using the ‘vegdist’ function in the R package *vegan* [66], specified with pairwise deletions to retain the zeros.

We used NMDS to express variation in two-dimensional space and validated the analysis by calculating the stress value (values smaller than 0.2 are acceptable; [67]). We further evaluated model fit using two methods: a goodness of fit plot of individuals, and a Shepard diagram of the linear and non-metric fit of the observed dissimilarities and their relation to the ordination distance.

We then overlaid the resultant NMDS cluster plot with the five elephant age classes to visually assess the relationship between age and relative position of individual character profiles. We used an Analysis of Similarities (ANOSIM) model to statistically test the difference between the five age classes. We used the ‘anosim’ function in the R package *vegan* [66] to fit each model with the dissimilarity matrix calculated for the NMDS model. The test statistic, *R*, is scaled from 1 to -1, where values greater than 0 means objects are more dissimilar between groups than within groups, and the opposite for values below 0 [67]. We considered groups to be significantly different from each other if the *R* value was greater than 0 and the corresponding *p*-value was significant.

## Results

### Social network centrality and dominance hierarchy analyses

**Fig 1:**
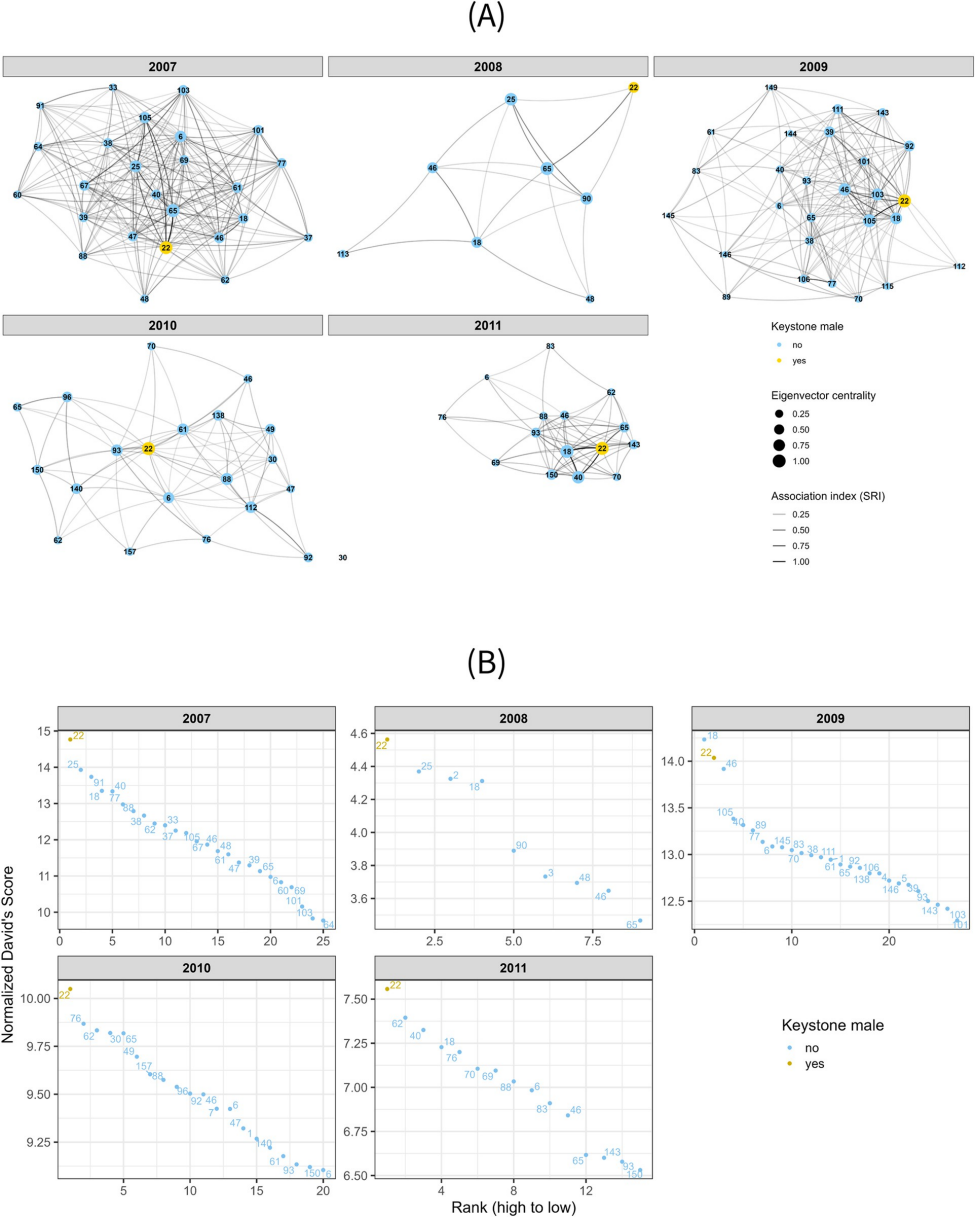
Social network and dominance hierarchy for each of the five years (2007–2011). The keystone individual (#22) is highlighted in yellow. (A) Social network centrality with a varied number of individuals per year (mean = 25, range = 8, 27). Circles represent individuals and the size of the circle represents how central they are to the network, binned into four levels. The lines between the individuals represent their association index relative to the individual the line is connecting to, where darker lines represent strong associations (also binned into four levels). (B) Dominance hierarchy with a varied number of individuals per year (mean = 25, range = 9, 27). Each point and corresponding number represent an individual. Due to differences in the number of individuals and David’s Scores in each year, both the x and y axes are displayed on different scales to better display the rankings.

The final number of individuals included in the annual social networks varied substantially after applying filtering criteria (mean = 25, range = 8, 27 individuals; Fig 1A). Our network analyses indicated that one individual, a full-sized adult male (Male #22) had the highest average eigenvector centrality of all individuals included in our analysis across all five years (mean = 0.91; SD = 0.18). This male was also the only full-size adult that was consistently sighted (i.e., observed at three or more independent observation events during a season) across all five years of the study.

The final number of individuals included in the annual displacement-based dominance hierarchies also had substantial variation and were composed of an average of 25 males of all age classes (range = 9, 27). The same individual identified as consistently having the highest eigenvector centrality (Male #22) also had the highest normalized David’s Score in all but one of the study years (2009) in which he had the second highest score, but with a very small difference between him and the highest-ranking adult male of 0.2 (Fig 1B).

Taken together, our analyses suggest that the same individual was consistently the most dominant and most central to the network of male elephants over the study period. This individual was then termed the keystone individual and was included as a social context to assess the impact he has on other males in the study.

### Behavioral repeatability

We found that five of the ten behaviors were significantly repeatable-by-individual (Table 3). All repeatabilities were low except self-directed comfort which had the highest repeatability estimate ± SE (*R* = 0.42 ± 0.10). Affiliation (*R* = 0.17 ± 0.05) and dominance (R = 0.17 ± 0.06) had the next highest repeatabilities, followed by aggression (*R* = 0.14 ± 0.05) and self-directed anxious (*R* = 0.14 ± 0.05).

**Table 3:** Repeatability estimates (individual, year [time], event [social context]) calculated for each behavior.

Behavior	n	Individual Repeatability			Year Repeatability			Event Repeatability		
		*R* ± SE	95% CI	*p*	*R* ± SE	95% CI	*p*	*R* ± SE	95% CI	*p*
Affiliation	34(408)	**0.17 ± 0.05**	**0.07, 0.28**	**< 0.001**	0.02 ± 0.03	0, 0.09	0.264	**0.21 ± 0.05**	**0.11, 0.31**	**< 0.001**
Aggression	34(429)	**0.14 ± 0.05**	**0.06, 0.23**	**< 0.001**	0.04 ± 0.04	0, 0.14	0.012	**0.27 ± 0.05**	**0.17, 0.38**	**< 0.001**
Dominance	30(310)	**0.17 ± 0.06**	**0.05, 0.30**	**< 0.001**	0.01 ± 0.02	0, 0.06	0.245	**0.15 ± 0.06**	**0.03, 0.27**	**0.006**
Escalated aggression	16(52)	0 ± 0.07	0, 0.23	1	0 ± 0.06	0, 0.22	1	0.36 ± 0.23	0, 0.75	0.082
Play	26(154)	0.10 ± 0.06	0, 0.23	0.009	0.03 ± 0.05	0, 0.16	0.29	**0.34 ± 0.10**	**0.12, 0.52**	**0.003**
Retreat	21(106)	0.01 ± 0.04	0, 013	0.412	0 ± 0.04	0, 0.13	1	**0.50 ± 0.13**	**0.20, 0.7**	**0.006**
Self-directed anxious	34(414)	**0.14 ± 0.05**	**0.05, 0.23**	**< 0.001**	0.08 ± 0.07	0, 0.24	< 0.001	**0.28 ± 0.06**	**0.18, 0.4**	**< 0.001**
Self-directed comfort	24(154)	**0.42 ± 0.10**	**0.20, 0.60**	**< 0.001**	0 ± 0	0, 0	1	0.03 ± 0.05	0, 0.18	0.355
Social contentment	32(362)	0.07 ± 0.03	0.01, 0.14	< 0.001	0 ± 0.2	0, 0.05	1	**0.46 ± 0.06**	**0.34, 0.57**	**< 0.001**
Vigilance	34(502)	0.05 ± 0.03	0.01, 0.11	< 0.001	**0.12 ± 0.08**	**0.001, 0.29**	**< 0.001**	**0.31 ± 0.06**	**0.21, 0.42**	**< 0.001**

Sample sizes (n) are presented as the number of individual elephants (number of behavioral rates). SE = standard error; CI = confidence interval. Significant repeatabilities are bolded.

Only vigilance behaviors showed a significant effect of year (R = 0.12 ± 0.08). For four of the five repeatable-by-individual behaviors (affiliation, aggression, dominance, self-directed anxious), event repeatabilities were significant and similar to, or higher than, the individual repeatabilities (Table 3). Four behaviors that were not significantly repeatable-by-individual were significantly repeatable-by-event: retreat had the highest event repeatability (*R* = 0.50 ± 0.13), followed by social contentment (*R* = 0.46 ± 0.06), play (*R* = 0.34 ± 0.10), and vigilance (*R* = 0.31 ± 0.06). Escalated aggression was not significantly repeatable for any of the random effects. However, the event repeatability was high at *R* = 0.36 ± 0.23, suggesting some impact of social context.

### Behavioral flexibility among social contexts

**Fig 2:**
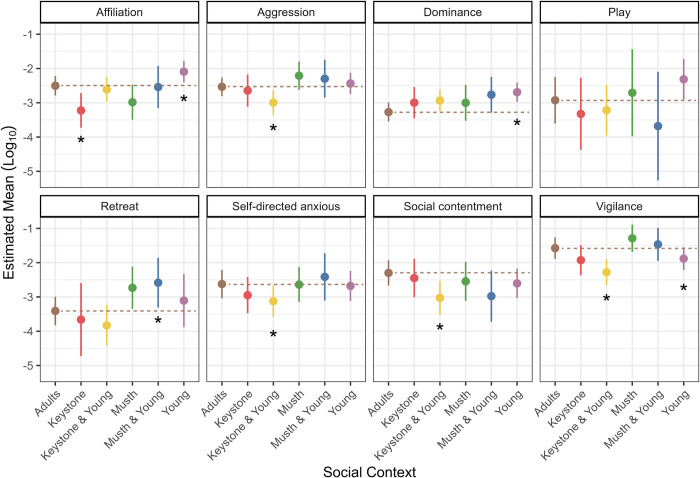
Marginal means for each of the eight behaviors with high event repeatability in six different social contexts. Only rates of adults (age classes 3Q, full, and elder) are included in this analysis. The social context refers to the presence of the keystone male, musth males, and young males, but does not include their behavior rates. The mean rates are displayed using the natural log transformation. Sample sizes and details of models are reported in S3 Table. Error bars represent the upper and lower 95% confidence intervals. Asterisks represent contexts that differ significantly from the “adults only” baseline category. The brown, dashed line represents the mean of the “adults only” category to allow for easier comparisons of where the means for the other social contexts fall in relation to this baseline category (i.e., higher or lower than the baseline).

We found that the presence of the keystone male and young males (age classes 1Q and 2Q) had the most impact on adult male elephant behavior amongst the repeatable-by-event behaviors (Fig 2; S3 Table). When the keystone male was present, the rate of affiliation behaviors significantly decreased (estimate = -0.72, SE = 0.27, p = 0.009). When young males were present, the rate of affiliation (estimate = 0.41, SE = 0.18, p = 0.021) and dominance (estimate = 0.58, SE = 0.16, p < 0.001) behaviors significantly increased, while the rate of vigilance behaviors decreased significantly (estimate = -0.31, SE = 0.13, p = 0.021). When both the keystone male and young males were present, significant decreases in aggression (estimate = -0.46, SE = 0.18, p = 0.014), self-directed anxious (estimate = -0.50, SE = 0.16, p = 0.003), social contentment (estimate = -0.73, SE = 0.22, p = 0.001), and vigilance (estimate = -0.70, SE = 0.16, p < 0.001) were observed. Finally, the rate of retreat behaviors increased slightly significantly when musth and young males were present (estimate = 0.82, SE = 0.41, p = 0.050).

### Age related differences in character profiles

**Fig 3:**
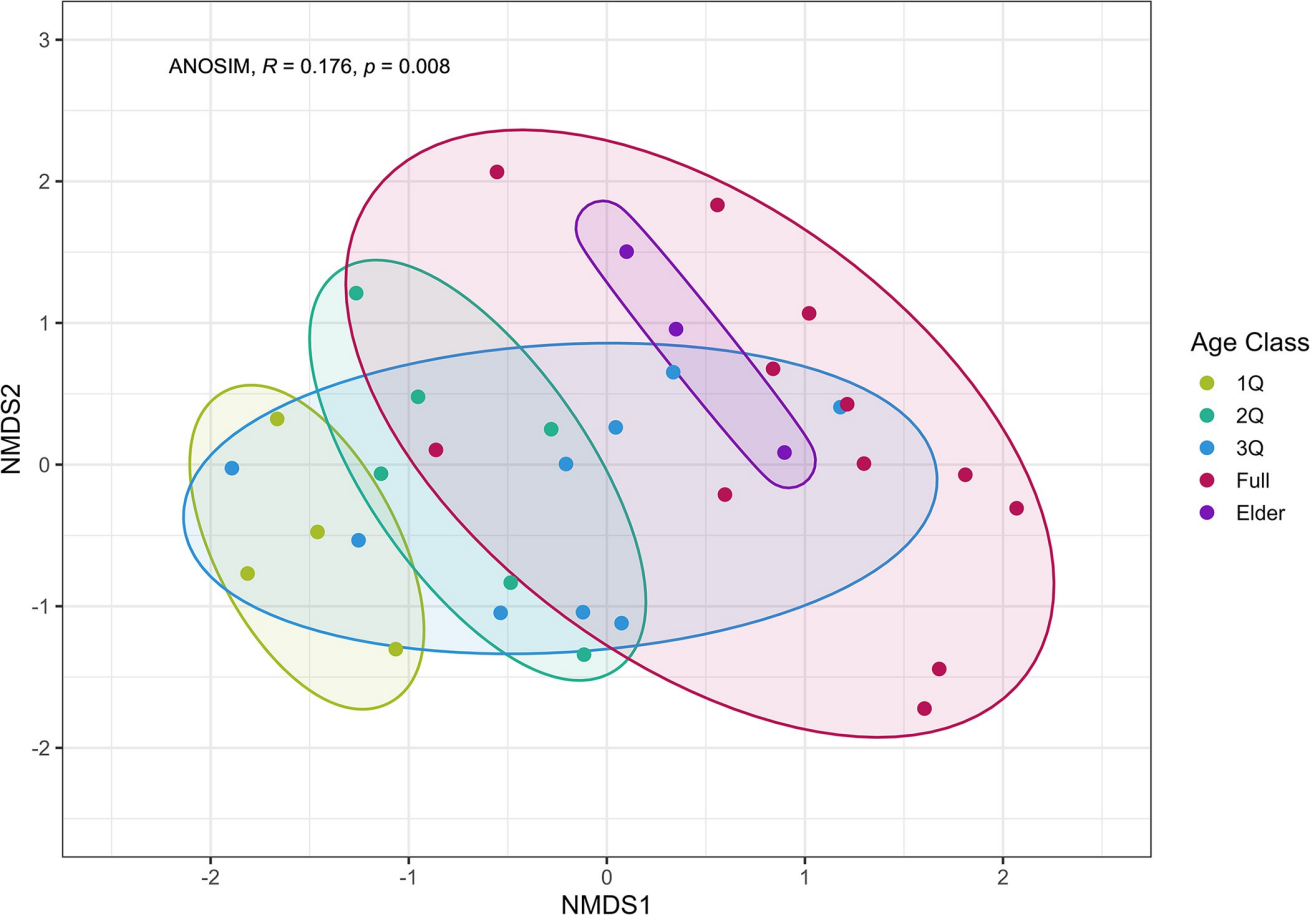
NMDS results when overlaid with the five age classes. Points represent individuals (n = 34), and the colors represent each age class (1Q, n = 4, 2Q, n = 6, 3Q, n = 9, full, n = 12, elder, n = 3). Rather than interpreting where points lie in relation to the axes, the points should be interpreted in relation to each other. Points, or individuals, that are close together have more similarities in character profiles, while individuals farther apart are more dissimilar. ANOSIM analysis revealed significant differences in character profiles between age classes (R = 0.176, *p* = 0.008).

Our NMDS analysis revealed that many of the 34 individuals display similar character profiles, while some individuals are dissimilar to most of their conspecifics (Fig 3, stress value of 0.08). The ANOSIM model indicated significant differences between the five age classes (*R* = 0.176, *p* = 0.008). 1Q, 2Q, and full-size males grouped closely within age class and occupied nearly separate areas on the figure, with the exception of one full size male grouping with the 2Q’s. Elder males grouped closely together and occupied a small area within the full-size male ellipse, suggesting similarities in character profiles among the elders. 3Q males were spread across the range of all other age classes, suggesting they display a variety of character profiles with similar attributes to the four age classes.

## Discussion

Our study demonstrates both the consistency and flexibility of male elephant behavior across social contexts and time, as well as age-related differences in character profiles. The patterns seen in many of these behaviors suggest that individuals consistently exhibit some behaviors differently than others but are also behaviorally adaptable, depending on the social situation. Keystone and young male presence had the biggest impact on adult males’ behavior rates across the repeatable-by-event behaviors. We show that the presence of these two social contexts had a positive effect on the psychological state of adult males, specifically increasing affiliative behaviors and reducing aggressive, anxious, vigilant, and social contentment behaviors. In contrast, social environments with musth males had little impact. Additionally, we found significant, age-related differences in character profiles for the behaviors that were consistent at the individual level, suggesting a large variation in the display of repeatable-by-individual behaviors. We discuss these results in the context of previous elephant character research, the life history and social dynamics of male elephants, and conservation and management implications.

### Behavioral consistency and flexibility

Five behaviors were repeatable-by-individual, which suggests that individual male elephants have distinct character traits (Table 3). These repeatable-by-individual behaviors are those in which individuals behave consistently across time and context, and are also different from each other [2]. As such, our results suggest that male elephants in this population are consistent both in the behaviors they initiate (affiliation, aggression, dominance) and how they respond to the social setting through self-directed behaviors (expressing behaviors that indicate anxiousness or comfort).

The consistent behaviors found in this study reflect both similarities and differences with previous studies of elephant character (or personality) of captive, semi-captive, and free-ranging elephants. Direct comparisons among studies are difficult to make, primarily due to differences in the behaviors assessed, how behaviors were defined, and the sex of the individuals included in the study. However, our results support previous findings of consistency in traits related to sociability and aggression [9–11, 14, 16–18, 22], suggesting the importance of these two traits for elephant character. These two traits are representative of the highly social nature of both males and females, as well as the aggression which occurs within dominance interactions between and within family groups [68–70], and between males [24].

The expression of affiliation and aggression behaviors in males appear to be correlated with position in the social network and dominance hierarchy. Highly socially-integrated males (such as #18, #22, #25, #46, and #65; Fig 1A) display higher and equal proportions of affiliation and aggression behaviors, and the most dominant males (such as #18, #22, #25, #40, and #62; Fig 1B) commonly display higher and equal proportions of affiliation and aggression (#2, #25), or aggression and self-directed anxious (#18, #40, #62) behaviors. This suggests that those that display equal amounts of affiliation and aggression behaviors are the most successful socially, as well as being more dominant. This finding matches our previous study, comparing behaviors across both wet and dry years over a four-year period between 2005–2008, where the most dominant individual (#22) displayed equal amounts of affiliation and aggression behaviors [24]. In our current study, we suggest that individuals who are both highly socially-integrated and dominant balance affiliation and aggression behaviors to maintain bonds as well as their position in the hierarchy. The interplay of dominance and affiliation behaviors is thought to facilitate bonds among individuals within social groups [71] and is the case here as well.

Repeatable-by-event behaviors are those which have rates that are stable within events and differ amongst events. While we expected more flexibility in some behaviors than others in order to cope with a dynamic environment [72], we did not expect four of the five of the repeatable-by-individual behaviors (affiliation, aggression, dominance, and self-direct anxious) to also be repeatable-by-event. Male elephants might have a ‘baseline’ level for each of these four behaviors that are also impacted by other factors, such as the social context or dyadic and group-level relationships. The overlap in individual and event repeatabilities for these four behaviors might be indicative of the ‘individual x environment’ interaction, where individuals are responding differently in the same context [2, 73] or have varying levels of behavioral flexibility [72]. This ‘individual x environment’ effect might be important to consider when conducting future elephant character research. Further, repeatability values do not provide insight into the degree of behavioral consistency of individuals, rather an overall population value of the individuals measured [2]. Thus, discerning which repeatable-by-individual behaviors are more susceptible to individual variation and social or environmental context is not possible with repeatability models.

Four behaviors were only repeatable-by-event (play, retreat, social contentment, and vigilance; Table 3). The low individual level repeatability suggests that individuals are more flexible in these behaviors which might be more susceptible to environmental perturbations than the behaviors that were repeatable-by-individual. For example, a behavior such as retreat might be more contextually dependent as it is often a response to aggression behaviors directed towards the focal subject. Similarly, play behaviors often decline with age in male elephants [26, 74] and are likely most often dependent on the presence of younger individuals [26, 75]. For all repeatable behaviors, a large proportion of the variance was unexplained, suggesting several other factors are likely contributing to aspects of behavioral consistency and flexibility in male elephants. Some of these variables might be environmental (e.g., time of day and rainfall conditions), physiological (e.g., hormone concentrations, body condition, and metabolic state), or genetic.

### The influence of social environment on adult male behavior

Given the reputation of musth males being highly aggressive, we expected their presence to have a larger impact on adult male behavior than we observed in this study. However, the presence of musth males only had a significant impact on one behavior—retreat (Fig 2, S3 Table). When musth and young males are present, the rate of retreat significantly increased for adult males, while just the presence of musth males increased retreat, vigilance, and aggression rates but not significantly. Male elephants rise in the dominance hierarchy when they are in musth [31, 76, 77]. Being reproductive competitors, non-musth, age-matched males are likely avoiding aggressive encounters with musth males. Additionally, aggression might be directed only towards individuals who are the highest ranking in the group, rather than blanket aggression towards all males [36]. Musth males might also be more inconsistent in their behavioral expression. In addition, character traits observed outside the state of musth might manifest, perhaps to a lesser degree, during musth. For example, those individuals who are less aggressive and more affiliative outside of musth, might have a different impact on conspecifics during musth than those who are more aggressive. Further research is needed to better understand the nuances of musth male behavior and the impact they have on conspecifics.

As we hypothesized, younger males (age classes 1Q and 2Q) impacted the behavior of adult males (Fig 2, S3 Table). Their presence significantly increased dominance behavior in adults. Here, and in a previous study [24], adult males were relatively stable in their dominance ranking, while younger males shifted in their position between years. This suggests younger males might be more actively trying to establish themselves in the hierarchy, and older males in the group might be increasing their expression of dominance behaviors to maintain their position while younger males are present.

We found the presence of young males caused an increase in affiliative behaviors in adults, which supports previous findings [26]. Given that affiliative behaviors strengthen bonds between individuals within social groups [71], our results suggest that mixed-age groups of male elephants are adaptive by enhancing group cohesion and improving the psychological state of older males. Another possible advantage of increased affiliative behaviors in adults might be to regulate hormones in younger males, which would reduce competition. Even just the presence of older males suppresses testosterone levels in younger males [78, 79], although not yet demonstrated, increased affiliation might further facilitate this suppression.

While we expected all social contexts to have an impact on adult male elephant behavior, we were not expecting the presence of both the keystone male and young males to significantly impact the expression of four of the eight behaviors (Fig 2, S3 Table). Their presence significantly reduced the expression of aggression, self-directed anxiety, vigilance, and social contentment (reduction in social contentment behaviors indicated a move from more passive to active behaviors). The keystone male and his close associates were observed”policing” aggression behavior in other males, particularly younger individuals, an observation that was also noted in another study system [29]. This reduction in aggression might explain the reduced vigilance and self-directed anxiety behaviors observed in the presence of the keystone male, whereby others may have felt less threatened, knowing that such policing would likely occur. Additionally, since the keystone male maintained his position in the dominance hierarchy throughout the study (Fig 1B), the hierarchy might be more structured overall when he is present, further reducing aggression and anxious behaviors related to uncertainty of position in the hierarchy.

### Age related differences in character profiles

The behavioral profiles of younger adult males (1Q and 2Q) display tight clustering within their age class (Fig 3), indicating that there is less variability in character profiles and personality structures in young males. Males in this age group are transitioning or have recently transitioned into independence and adulthood, exploring away from their family group’s range [32]. On the younger side of this age range, survival is challenging, and only about half of African elephant males survive to 30–35 years old [80]. The tight clustering of behavioral profiles for these age classes suggests limited behavioral strategies to increase chances of survival. Not only are young males seeking out food and water sources, but they also face a drastic change in their social lives, integrating from a predominantly female and family-based life to adult male elephant society [32]. Young males are thought to be forming relationships with peers and older adults to gather environmental knowledge and social skills [26, 27, 30, 32]. These males are also beginning to integrate into local dominance hierarchies, where size and therefore age are important factors in determining position [24]. As such, these males are likely more constrained in how they display these repeatable-by-individual behaviors. However, it remains unclear whether constraint on behavior might create more stability for the duration of the individual’s time in these age classes, or if more flexibility is required to adapt to the changing environment.

After transitioning into independence, adult male elephants might have more freedom in their behavioral expression, as indicated by the large variation in character profiles for the 3Q, full, and elder groups (Fig 3). The social niche specialization hypothesis posits that variation in behavior reduces direct competition between individuals [1, 81]. For male elephants, intra-specific competition is high, but the adoption of social niches, further facilitated by the maintenance of relatively stable dominance hierarchies [24], might help to reduce conflict. Further, social niches might play a stronger role in species that maintain group membership than species with lower levels of repeated interactions [1]. Our results support this idea, since the individuals included in this study are those who are the most frequent visitors to Mushara, where interactions amongst these individuals are common. Despite apparent social niches in adult male elephants, some individuals are grouped very closely together, suggesting a possible effect of behavioral cohesion amongst individuals with higher association indexes. From our results, age class appears to contribute in large part to driving social niches and possibly behavioral cohesion, but future research is needed to tease apart the impact that age, genetics, association, and dominance have on group dynamics.

### Conservation Implications

Older males play an important regulatory role in all-male elephant populations, by maintaining social cohesion [25], mediating aggression behaviors [29, 78, 79], and functioning as a source of ecological information, by providing knowledge of how to effectively navigate through the environment [28, 30]. In our study we focused on the importance of younger males in male elephant society by demonstrating their positive influence on the psychological state of older males. Here and in a recent study, we found the keystone male and other mature, socially-integrated males to be important for group cohesion [28], further emphasizing the importance of mixed-age groups in any population. Given these findings, male elephant management practices should incorporate a healthy age distribution within a holistic approach to their conservation and management.

## Conclusions

This unique study offers critical insights into the individuality and sociality of free-ranging male elephants. Our results offer a blueprint to establish important aspects of male elephant character, and when combined with further research, may aid in improving conservation policies for male elephants *in-situ* and inform management decisions *ex-situ*. For example, elephant managers in captive settings could consider pairing or grouping appropriate individuals to offer social enrichment based on character traits and prioritize mixed-age groups and individuals who might take on a more active role in mediating relationships amongst group members. *In-situ* managers could use character research to predict the success rate of the increasingly more common translocations [82] or orphan releases [83, 84], and correlate character traits with the propensity to engage in risky foraging behaviors.

As human-accelerated climate change, shrinking habitats, poaching, and increased human-elephant conflict continue to put pressure on elephants, understanding the relationship between elephant character, and responses to environmental and social change are critical. Additionally, this research could aid in the development of human-elephant coexistence strategies by accounting for the inter- and intra-individual variation of elephants [85]. Finally, character research furthers our understanding of the complexity of male elephant social dynamics as they adapt to the changing world, and we can use this knowledge to make more informed conservation management decisions.

## Supporting information

S1 Table Overview of the total number of events, intervals, and observation time, as well as the number of individual elephants observed for each year. Since individual elephants were observed within two or more years, the total elephant number is for unique individuals across the study period.
(DOCX)

S2 Table Detailed version of the ethogram describing the ten behavioral categories and sixty-six discrete behaviors that make up the behavioral categories. Behaviors and categories are adapted and modified from [24, 41]. For behavior categories, n represents the total occurrence of discrete behaviors within that category, and for discrete behaviors, n represents the total number of times the behavior was displayed. All n values are pooled across individuals, events, and years.
(DOCX)

S3 Table Results of linear mixed models for each behavior that had a high event repeatability. Sample sizes (n) are presented as the number of individual elephants (number of behavioral rates). All models included a nested random effect of year, event, and intervals, as well as individual elephant ID as a partially-crossed random effect, with social context as a fixed effect, and the log of the behavior rate as the response variable. Only the behavior rates for non-keystone and non-musth older adults (age classes 3Q, full, and elder) were included in these analyses. The ‘adults’ social context was the reference category for all models, so *t* and *p*-values are not provided. Significant social contexts are highlighted with an asterisk. SE = standard error.
(DOCX)
