# Supplementary material for: Consistency and flexibility of character in free-ranging male African elephants across time, age, and social contexts
Source: PLoS One. 2024 Dec 4;19(12):e0311780. doi: 10.1371/journal.pone.0311780 (PMC11616828; doi:10.1371/journal.pone.0311780)
Supplement: S1 Table — Since individual elephants were observed within two or more years, the total elephant number is for unique individuals across the study period. (DOCX) [file pone.0311780.s001.docx]

| **Year** | **Events** | **Intervals** | **Total observation time (minutes)** | **Elephants (n)** |
| --- | --- | --- | --- | --- |
| 2007 | 37 | 47 | 3,124.5 | 23 |
| 2008 | 17 | 25 | 1,186.4 | 14 |
| 2009 | 40 | 59 | 2,794.6 | 28 |
| 2010 | 34 | 41 | 2,310.4 | 23 |
| 2011 | 20 | 28 | 964.6 | 18 |
| *Total* | *148* | *200* | *10,380.5* | *34* |

**S1 Table. Overview of the total number of events, intervals, and observation time, as well as the number of individual elephants observed for each year.** Since individual elephants were observed within two or more years, the total elephant number is for unique individuals across the study period.
